# Reliability and Construct Validity of Two Versions of Chalder Fatigue Scale among the General Population in Mainland China

**DOI:** 10.3390/ijerph13010147

**Published:** 2016-01-21

**Authors:** Meng-Juan Jing, Wei-Quan Lin, Qiang Wang, Jia-Ji Wang, Jie Tang, En-She Jiang, Yi-Xiong Lei, Pei-Xi Wang

**Affiliations:** 1 Institute of Public Health, School of Nursing, Henan University, Kaifeng 475000, China; jing53905@163.com (M.-J.J.); kfwq@henu.edu.cn (Q.W.); esjiang@henu.edu.cn (E.-S.J.); 2 Department of Preventive Medicine, School of Public Health, Guangzhou Medical University, Guangzhou 510182, China; linweiquan0503@163.com (W.-Q.L.); wjiaji@163.com (J.-J.W.); gytanjie@163.com (J.T.); gz-leizeng@163.com (Y.-X.L.)

**Keywords:** chalder fatigue scale, reliability, construct validity, exploratory structural equation modeling, confirmatory factor analyses, mainland China

## Abstract

The 14-item Chalder Fatigue Scale (CFS) is widely used, while the 11-item version is seldom to be found in current research in mainland China. The objectives of the present study is to compare the reliability and construct validity between these two versions and to confirm which may be better for the mainland Chinese setting. Based on a cross-sectional health survey with a constructive questionnaire, 1887 individuals aged 18 years or above were selected. Socio-demographic, health-related, gynecological data were collected, and 11-item and 14-item Chalder Fatigue Scale (CFS) were used to assess fatigue. Confirmatory factor analysis and exploratory structural equation modeling (ESEM) were performed to test the fit of models of the two versions. Confirmatory factor analysis of the two versions of CFS did not support the two-factor theorized models. In addition, a three-factor ESEM model of the 11-item version, but not the 14-item version, showed better factor structure and fitness than the other models examined. Both the versions had good internal consistency reliability and a satisfactory internal consistency (Ω = 0.78–0.96, omega coefficient indicates the internal consistency reliability) was obtained from the optimal model. This study provided evidence for satisfactory reliability and structural validity for the three-factor model of the 11-item version, which was proven to be superior to the 14-item version for this data.

## 1. Introduction

Fatigue is a sense of tiredness and weakness caused by a variety of reasons. Fatigue is not only a common symptom among patients with physical and mental diseases [1,2,3,4,5], but also one of the main complaints in the general population [6,7]. Considering the subjectivity of fatigue, accuracy on assessment of fatigue is increasingly important, so it is necessary to develop an accurate and valid assessment instrument, yet there are no indigenously developed tools to assess the fatigue for the general population in China, except for the tools directly imported from Western countries.

The Chalder Fatigue Scale (CFS), a brief and useful instrument, is one of the scales used frequently. The 14-item fatigue scale as a self-rating scale was developed to measure the severity of fatigue by Chalder and colleagues [8]. The related research has demonstrated its good validity and internal reliability. Repetitive verifications were conducted by researchers [9,10,11,12], and the positive results facilitated the wide application of the Chalder Fatigue Scale. It has been used to assess the symptom severity, screen fatigue cases in epidemiological studies [8], as well as estimate the treatment outcomes for fatigue [13,14,15]. It has been applied in not only patients with chronic fatigue syndrome [9], cancer [13], multiple sclerosis [16] and so on, but also in the general population [17].

Fourteen questions of the CFS were generated by various experts in this field, mainly divided into two dimensions, physical fatigue and mental fatigue. The revised 11-item scale was conducted based on the 14-item version with three items dropped. It was confirmed that, despite its brevity, the revised 11-item scale was still reliable and valid [8]. Subsequently, Morriss *et al.* [9] examined the application of the 14-item fatigue scale in patients with chronic fatigue syndrome and supported the validity of the 11-item version. In some areas such as Hong Kong and Brazil, the 11-item version is of good reliability and validity [10,12]. Additionally, the later study demonstrated that the 11-item version is better in terms of data-model fitting, so it is more commonly adopted in studies of fatigue [10]. However, as is known, in mainland China, the 14-item Chalder Fatigue Scale, abbreviated as FS-14, is more widely used than the 11-item version to assess fatigue [18,19,20,21,22]. It is worthy to identify whether the revised 11-item version will be as reliable as or maybe much better than FS-14 in the case of the Chinese mainland.

Although two factors were obtained in the original scale and revised version [8,12], three and four factors were more favored in other studies [9,23,24]. This diversity may correlate with different study populations and culture backgrounds. Given fewer studies about the structural exploration of CFS were conducted in mainland China, it is necessary to explore the factor structure of CFS in the mainland Chinese setting.

In this study, we carried out this investigation to compare the internal consistency and construct validity between these two versions to confirm which is better for the case of the Chinese mainland.

## 2. Methods

### 2.1. Sampling and Participants

**Figure 1:**
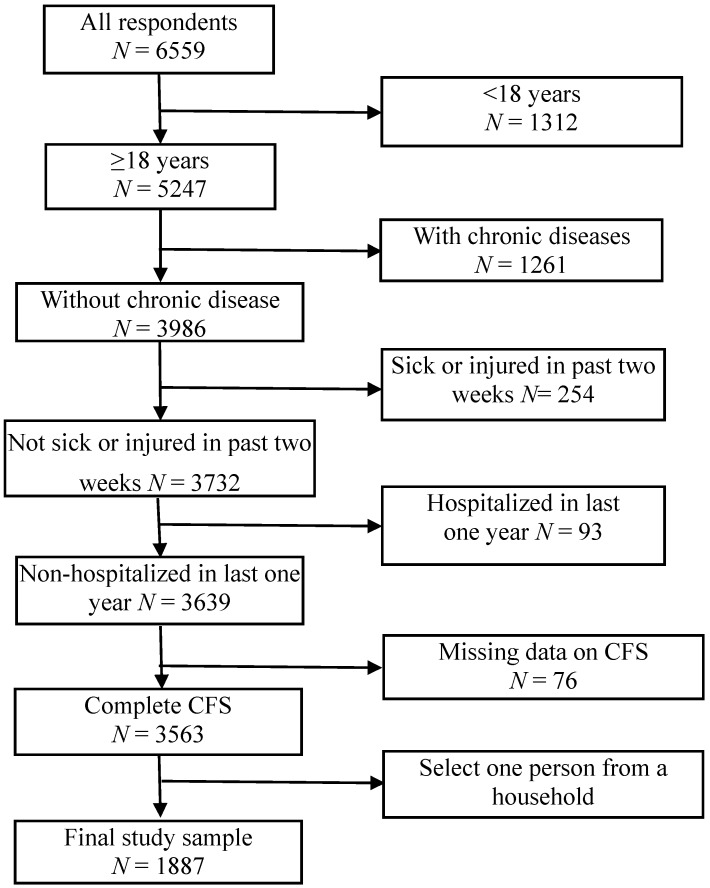
Flow chart in the selection of study subjects.

This study was based on a cross-sectional health survey in the Shunned municipality of Guangdong province in China. The sample in this survey consisted of family members drawn from 5% of total households in this municipality. A total of 2080 households, including 6802 residents, were randomly selected using the city’s household registration system via a simple random sampling method. A total of 243 individuals refused participation or did not respond, meaning that 6559 individuals took part in this survey. Ethical approval for this survey was obtained from the Research Ethics Board of Guangzhou Medical University. In addition, written informed consent was obtained from each participant prior to survey recruitment. For this study, we recruited individuals aged 18 years or above from all respondents except the cases with chronic diseases, sick or injured in the past two weeks or hospitalized in last one year. Given the non-independence of observation due to cluster sampling, we selected only one person from a household randomly, and, finally, 1887 adults were included in this analysis. A flowchart illustrating the selection of study participants is presented in Figure 1.

### 2.2. Procedures

The interviewers (medical students from Guangzhou Medical University and Guangzhou Pharmacy College, staff from local Community Health Service Agencies) underwent a survey-specific training, including the introduction of the survey, questionnaire content, confidentiality and communication skills. Interviewers were provided with full sets of written instructions of the data collection and recording procedures. Interviews took place in participants’ homes. Each interview group comprised of at least two medical students and a nurse or a physician, and the interview is mainly conducted face-to-face using structured study questionnaires. During the early stage of the study, each group was administered by a supervisor to ensure that all interviews were conducted correctly. Subsequently, the routine supervision was randomly performed in a certain group.

### 2.3. Fatigue Measurement

The Chinese mainland version of the Chalder Fatigue Scale, namely, FS-14 is translated from the original Chalder Fatigue Scale, comprised of 14 questions. Total fatigue score is calculated by considering all of the items and score the fatigue scale on a two-point scale (presence or absence) rather than the four-point Likert-type scoring. We graded 11 items using the positive scoring system with 1 = Yes, 0 = No, while Item 10, Item 13 and Item 14 (content of the scale was listed in the Table 1) were scored reversely. The revised 11-item fatigue scale is derived from the 14-item version by excluding Item 5, Item 10 and Item 14 [9,10,12]. The method of scoring for the 11-item version is the same as the 14-item version. 

**Table 1:** The questions of Chalder Fatigue Scale (CFS).

ITEM	Question
Item 1	Do you have problems with tiredness?
Item 2	Do you need to rest more?
Item 3	Do you feel sleepy or drowsy?
Item 4	Do you have problems starting things?
Item 5	Do you start things without difficulty but get weak as you go on?
Item 6	Are you lacking in energy?
Item 7	Do you have less strength in your muscles?
Item 8	Do you feel weak?
Item 9	Do you have difficulty concentrating?
Item 10	Do you think as clearly as usual?
Item 11	Do 6 you make slips of the tongue when speaking?
Item 12	Do you find it more difficult to find the correct word?
Item 13	Is your memory as good as usual?
Item 14	Are you still interested in the things you used to do?

### 2.4. Statistical Analysis

All statistical analyses were completed by Mplus 7.0 and SPSS version 13.0. Whereas the items of Chinese CFS applied were binary variables, traditional exploratory factor analysis (EFA) and confirmatory factor analysis (CFA) provided by AMOS or LISERL was inappropriate. Thus, models were examined by CFA and exploratory structural equation modeling (ESEM) with Mplus, using weighted least squares with adjusted mean and variance (WLSMV), which is a robust method for binary variables [25].

First, the theoretic two-factor model, namely physical fatigue composed of Item 1 to Item 8 and mental fatigue composed of Item 9 to Item 14, of the two versions were tested by CFA. In addition, ESEM was conducted to explore optimal models for the two versions. We then tested the association latent variables and covariates by adding gender, age, marital status, education and occupational status to the ESEM model. Finally, bifactor models were tested for the theoretic two-factor models of both versions to compare the reliability and the larger explained common variance (ECV) of the common factor, the better internal consistency of the scale [26]. Additionally, omega coefficient of the selected model was used to test the internal consistency reliability of sub-scale and total scale, with Ω ≥ 0.70 is considered acceptable [27]. The model fit was evaluated by the indices of goodness-of-fit, such as comparative fit index (CFI), Tucker–Lewis index (TLI), root mean square error of approximation (RMSEA), weighted root mean square residual (WRMR) without report of Akaike information criterion (AIC) and Bayesian information criterion (BIC) in the binary data. In the structural equation model of categorical variable, the model is considered to fit well with the data, as CFI > 0.95, TLI > 0.95, RMSEA < 0.06 [28], and WRMR ≤ 1.0 [29]. For comparison of nested models, given the type of our variables, DIFFTEST command provided especially by Mplus was submitted in the ESEM models. In addition, diversity between two models are considered to be significant, if the variance of the CFI and TLI free from sample size is larger than 0.01 [30]. The other statistical analysis was performed using SPSS 13.0.

### 2.5. Ethical Approval

Ethical approval for this survey was obtained from the Research Ethics Board of Guangzhou Medical University (Project identification code: 2014024). All procedures performed in studies involving human participants were in accordance with the 1964 Helsinki declaration and its later amendments.

## 3. Results

### 3.1. Description of the Samples

Ethical approval for this survey was obtained from the Research Ethics Board of Guangzhou Medical University. All procedures performed in studies involving human participants were in accordance with the 1964 Helsinki declaration and its later amendments.

### 3.2. Construct Validity

First, the original two-factor model, namely physical fatigue composed of Item 1 to Item 8 and mental fatigue composed of Item 9 to Item 14, was confirmed by CFA. To optimize the models, modification was carried out according to the residual correlation between Item 1 and Item 2 (Modification Index = 123.712) in the 14-item version, while Item 6 and Item 7 (Modification Index = 100.459) in the 11-item version. As shown in Table 2, the revised model of the 11-item version (CFI = 0.960, TLI = 0.948, RMSEA = 0.067, WRMR = 2.116) was no better than that of the 14-item version (CFI = 0.955, TLI = 0.945, RMSEA = 0.060, WRMR = 2.114). Overall, the original two-factor model of the two versions was not the ideal model to the data analyzed. Therefore, further analysis was conducted by exploratory structural equation modeling.

**Table 2:** The two-factor models of confirmatory factor analysis (CFA) for the two version.

Model	*χ*^2^	*df*	CFI	TLI	RMSEA (90% CI)	WRMR
14-version						
Original model	690.392 *	76	0.946	0.935	0.065 (0.061–0.070)	2.329
Revised model	587.572 *	75	0.955	0.945	0.060 (0.056–0.065)	2.114
11-version						
Original model	484.801 *	43	0.950	0.936	0.074 (0.068–0.080)	2.378
Revised model	396.625 *	42	0.960	0.948	0.067 (0.061–0.073)	2.116

* *p* < 0.05; CFA: confirmatory factor analysis, df: degree of freedom, CFI: comparative fit index, TLI: Tucker-Lewis index, RMSEA: root mean square error of approximation, CI: confidence interval, WRMR: weighted root mean square residual.

The results of ESEM were shown in the Table 3. For the 14-item version, compared with the recognized two-factor model, the fit indices of the two ESEM models were improved significantly. Though the two-factor ESEM model was further improved with the modification of residual correlation between Item 13 and Item 14 (MI = 97.987), the factor loading matrix indicated that the loading of Item 14 was lower than 0.3 on either of the two factors. For the three-factor ESEM model, the fit indices were best, but its structure was unsatisfactory. Only Item 13 was significantly loaded on the third factor, moreover Item 10 and Item 14 had non-significant loading on any of these factors. Thus, the models of the original version were unacceptable.

**Table 3:** The two- and three-factor models of exploratory structural equation modeling (ESEM) for the two versions.

Model	*χ*^2^	*df*	CFI	TLI	RMSEA (90% CI)	WRMR
14-version						
Two-factor	351.972 *	64	0.975	0.964	0.049 (0.044–0.054)	1.483
Revised Two-factor	255.833 *	63	0.983	0.975	0.040 (0.035–0.045)	1.249
Three-factor	208.555 *	52	0.986	0.976	0.040 (0.034–0.046)	1.074
11-version						
Two-factor	129.677 *	34	0.989	0.983	0.039 (0.032–0.046)	1.057
Revised Two-factor	67.444 *	33	0.996	0.994	0.024 (0.015–0.032)	0.741
Three-factor	33.979	25	0.999	0.998	0.014 (0.001–0.025)	0.474
Three-factor + covariates	142.419 *	65	0.991	0.985	0.025 (0.020–0.031)	0.864

* *p* < 0.05; ESEM: exploratory structural equation modeling, df: degree of freedom, CFI: comparative fit index, TLI: Tucker-Lewis index, RMSEA: root mean square error of approximation, CI: confidence interval, WRMR: weighted root mean square residual.

For the 11-item version, either the two- or the three-factor ESEM model was better than the theoretic two-factor model. In contrast to the revised two-factor ESEM model with the modification of residual correlation between Item 11 and Item 12 (MI = 63.062), the three-factor model seemed to be fitter to the data (CFI = 0.999, TLI = 0.998, RSMEA = 0.014, WRMR = 0.474) without modification indices above the minimum value 10.0. To determine which model is better for this data, the Chi-Square test for difference testing was used, and the definite differences (Δ*χ^2^* = 28.708, Δ*df* = 8, *p* < 0.001) implied that the three-factor model was superior. According to the results in Table 4, Item 1 to Item 3 composed factor 1 (General feeling for fatigue), Item 11 and Item 12 were separated from the two-factor model as factor 3 (Language difficulties), and the rest composed factor 2 (Specific feeling for fatigue). Obviously, given the structure and fitness of model, the three-factor ESEM model of the 11-item version is appropriate to the data.

**Table 4:** Factor loading on the three-factor ESEM models of the FS-11.

Item	Factors		
	General Feeling for Fatigue	Specific Feeling for Fatigue	Language Difficulties
Item 1	0.989 *	0.005	−0.127
Item 2	0.962 *	−0.041	−0.001
Item 3	0.859 *	0.060	0.031
Item 4	0.361 *	0.549 *	0.002
Item 6	0.096	0.822 *	0.016
Item 7	−0.010	0.916 *	−0.061
Item 8	0.244 *	0.446 *	0.211 *
Item 9	0.092	0.512 *	0.231 *
Item 11	−0.137	0.003	0.934 *
Item 12	0.002	0.176	0.733 *
Item 13	0.090	0.555 *	−0.166

* *p* < 0.05; ESEM: exploratory structural equation modeling, FS-11: 11-item of the Chalder Fatigue Scale.

**Figure 2:**
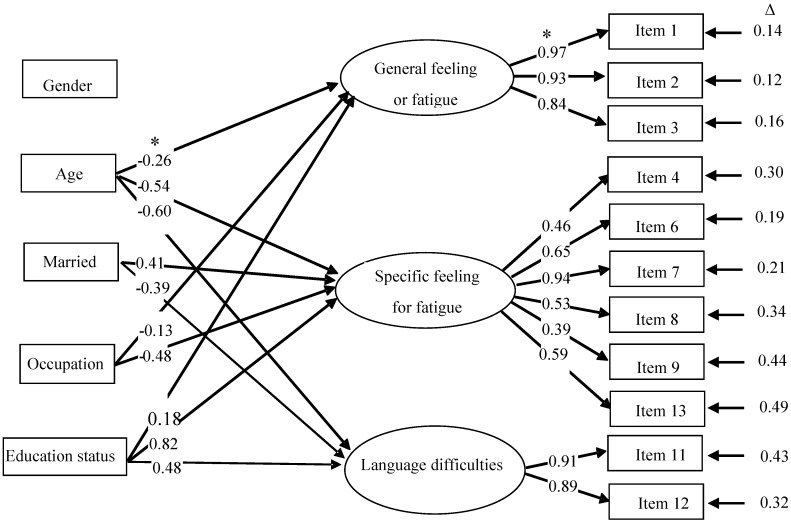
Associations between the CFS factors and covariates in the three-factor ESEM model of 11-item version. * Correlation coefficients; Δ residual error.

The three-factor ESEM model with covariates of the 11-item version provided a satisfactory fit to the data (CFI = 0.991 TLI = 0.985, RMSEA = 0.025, and WRMR = 0.864). The associations between the latent factors and the covariates are presented in Figure 2. Expect for gender, correlation coefficients were significant between age, marital status, education and occupational status and the three latent variables (absolute *r* values = 0.13–0.82, *p* < 0.05).

### 3.3. Internal Consistency

Though the value of ECV in the 14-item version was higher than that of the 11-item version (76.8% *vs*. 72.2%), both of them had good internal consistency reliability. For the optimal model, the three-factor model of the 11-item version, the omega coefficient was 0.89 for general feeling for fatigue, 0.782 for specific feeling for fatigue, 0.96 for the language difficulties, and 0.84 for the total stale.

## 4. Discussion

Originally, two principal components of fatigue were obtained from the 14-item fatigue scale by Chalder *et al.* [8], and the 11-item fatigue scale by Cho *et al.* [12] in the general practice sample. However, the original two-factor model, either the 14-item version or the 11-item version, failed to fit our data by confirmatory factor analysis despite the good internal consistency reliability. That might be cultural diversity, which makes individuals express emotions in different ways that resulted in the different structures of CFS.

To explore the optimal model to the present general population, we examined different structural models for the 14-item and the 11-item version based on exploratory structural equation modeling. For the 14-item version, the factor loading of Item 14 was lower than 0.3 in the two- and the three-factor models, and factor loadings of Item 10 and Item 14 were lower than 0.3 in the three-factor models. It supported the rationality to remove items from the original version. For the 11-item version, the two-factor ESEM model and the three-factor ESEM model are both appropriate for this data. Comparing the two models, we found the fit indices of the three-factor model were better than that of the two-factor model. In the three-factor model of the 11-item version, most of the included items loaded strongly onto only one of the factors of fatigue. Morriss *et al.* [9] and Yang *et al.* [24] obtained four constructs of fatigue, which were different from ours. A probable interpretation for these discrepant findings may lie in different methods of analysis, or partly due to the variance from population investigated and sample size. Recently, a study from Hong Kong [23] showed evidence that a three-factor model of the 11-item CFS using ESEM provided a good fit to their data. The three factors consisted of physical fatigue (problems with tiredness, more rest, feeling sleepy or drowsy), low energy (problems starting things, lack of energy, less strength in muscles, feeling weak, having trouble concentrating) and mental fatigue (hard to concentrate, making slips of the tongue, hard to find the correct words, poor memory). In contrast to their results, we obtained three factors from the 11-item version, but the content of each factor is different. In the context of traditional culture, the way people express their feelings is more implicit. Especially Chinese on the Mainland tend to confuse the physical and mental symptoms when expressing feelings of fatigue. That might interpret the reason that there is no clear demarcation between physical fatigue and mental fatigue, which is different from previous studies. The method of scoring the fatigue scale is two-point (presence or absence) rather than the four-point Likert scoring created a lack of precision in expression of feelings.

In the model with covariates, expect gender, age and marital status, occupation and education status could foreshadow the level of fatigue, which could provide information to prevent accidents from fatigue.

Comprehensively, factor structure and the fit indices indicated that the 11-item version was significantly better than the 14-item version for the studied sample, which was consistent with another study from Hong Kong [10].

Although this study may be limited to the healthy population in the community in Mainland China, it could add knowledge on fatigue evaluation and supply an important reference for fatigue research.

### Strengths and Limitations of Our Study

As we know, this is the first study to provide evidence to examine the reliability and construct validity of the 14-item version and the 11-item version of CFS for a large sample in mainland China. However, the study has certain limitations. Since this health survey is not specifically designed for this study, we did not take into account the family clustering of data. Therefore, the discriminate validity of CFS was not tested due to the absence of data on anxiety and depression of the interviewees. Finally, the sample does not include people with illness and hospitalization, not supporting the generalizability of findings. Further studies may be need to provide more confirmation from other groups of people.

## 5. Conclusions

Our study is the first study to compare the reliability and construct validity of the 14-item version and the 11-item version of CFS in mainland China. The study proved that CFS is a reliable and valid instrument for assessing fatigue among the general population in mainland China. The achieved results also confirmed that the 11-item version was superior over the 14-item version in terms of data-model fitness in the Chinese general population.
